# Mapping health-related quality of life measurement tools in Africa: a scoping review

**DOI:** 10.1186/s41687-026-01100-6

**Published:** 2026-05-30

**Authors:** Begashaw Melaku Gebresillassie, Lucky Gift Ngwira, Jermaine Dambi, Adeladlew Kassie Netere, Eyayaw Ashete Belachew, Abdelghafour Marfak, Jaafar Chemli, Anthony Waka Udezi, Girma Tekle Gebremariam, Ian Neethling, Samar Farid, POPQOL Africa Advisory

**Affiliations:** 1 Calvary Mater Newcastle Hospital, Waratah, Australia; 2 University of Newcastle Australia, Newcastle, Australia; 3 Kamuzu University of Health Sciences, Blantyre, Malawi; 4 University of Zimbabwe, Harare, Zimbabwe; 5 Monash University, Melbourne, Australia; 6 University of Tasmania, Hobart, Australia; 7 National Health of Public Health, Rabat, Morocco; 8 Tunis El Manar University, Tunis, Tunisia; 9 University of Benin, Benin City, Nigeria; 10 Addis Ababa University, Addis Ababa, Ethiopia; 11 South African Medical Research Council, Cape Town, South Africa; 12 Cairo University, Giza, Egypt; 13 EuroQol Research Foundation, Rotterdam, Netherlands

**Keywords:** Africa, EQ-5D, WHOQOL-BREF, SF-36, Health-related quality of life, Health technology assessment, Patient-reported outcome measures

## Abstract

**Background:**

Health-related quality of life (HRQoL) is an important measure in healthcare that captures the multidimensional effects of health on individuals’ lives. While HRQoL measures are widely used globally, there is limited consolidated evidence of their application in Africa. This review aimed to explore and map the available evidence on the utilization of HRQoL measures in Africa, with a focus on understanding their application across diverse populations, health conditions, and geographical regions while identifying gaps, methodological inconsistencies, and opportunities for future research.

**Methods:**

The scoping review was conducted following the Arksey and O’Malley framework. Electronic databases, including Medline, Embase, Emcare, CINAHL, and Cochrane Library, and other sources, were searched from the inception up until August 2025. Results were synthesized narratively with descriptive statistics, presented using tables and graphs.

**Results:**

Out of 20,438 records identified, 731 studies met the inclusion criteria. The geographical distribution revealed significant disparities, with over half of the studies originating from Nigeria (21.8%), Ethiopia (14.8%), and South Africa (14.5%). Most studies (74.2%) employed descriptive designs and were conducted in hospital settings (70.3%). HRQoL instruments were used across a wide range of health conditions, with HIV/AIDS (20.5%), cancer (10.1%), and diabetes (7.1%) being the most frequently studied. Generic HRQoL tools such as WHOQOL-BREF (19.7%), SF-36 (16.4%), and EQ-5D (13.1%) were the most commonly utilized, accounting for nearly half of all studies. The analysis of information reporting practices for EQ-5D instruments revealed inconsistencies, with only 30.2% of studies reporting all three components (descriptive profile, index scores, and EQ-VAS).

**Conclusion:**

This review highlighted the increasing use of HRQoL measures in African health research over the past two decades while identifying significant gaps. Most studies were descriptive, concentrated in a few countries, and primarily conducted in healthcare settings, limiting broader applicability. The frequent use of generic HRQoL instruments underscores their importance and cultural adaptation. Additionally, inconsistencies in reporting practices and methodological limitations hinder the comparability and utility of findings. To advance HRQoL research and its application, it is essential to address these gaps through standardized guidelines, broader geographical representation, and improved methodological rigor.

**Supplementary Information:**

The online version contains supplementary material available at 10.1186/s41687-026-01100-6.

## Background

Health-related quality of life (HRQoL) has emerged as an important outcome measure in healthcare research and practice [1]. For the purposes of this review, HRQoL is operationalized as the health-related component of quality of life, reflecting individuals’ perceived physical, psychological, and social functioning and well-being as affected by health conditions, symptoms, treatment, and healthcare [1]. This distinguishes HRQoL from the broader concept of quality of life (QoL), which may also encompass wider personal, social, cultural, economic, and environmental aspects of life [1]. HRQoL offers a comprehensive assessment of patients’ well-being, encompassing physical, mental, and social domains [1]. By focusing specifically on the impact of health and healthcare on daily functioning and perceived well-being, HRQoL provides valuable insights from the patient’s perspective [2].

In recent decades, there has been a growing recognition of the importance of HRQoL in informing clinical decision-making, healthcare policy, and resource allocation [3]. Also, HRQoL measures are increasingly utilized in clinical trials, observational studies, and health technology assessments to evaluate the effectiveness of interventions, compare treatment options, and guide patient-centered care [4]. Health technology assessments, in particular, have become indispensable tools for systematic healthcare priority setting, especially in resource-constrained settings like Africa [5]. Institutionalization efforts, supported by initiatives such as the Africa CDC’s Health Economics Program, aim to promote evidence-based decision-making amidst the continent’s dual challenge of a high disease burden and limited health budgets [5]. In this context, HRQoL measures, especially preference-based measures, such as the widely used EuroQol five-dimension (EQ-5D) instrument, play a pivotal role in evaluating the cost-effectiveness of interventions and generating quality-adjusted life years (QALYs) to inform resource allocation [6].

Africa faces a complex landscape of health challenges, including infectious diseases like HIV/AIDS and malaria, the rising prevalence of non-communicable diseases (NCDs) such as diabetes, mental disorders, among others [7]. The burgeoning dual burden of infectious diseases and NCDs is further exacerbated by socio-economic disparities [7, 8]. This dual epidemic contributes to significant morbidity and mortality, underscoring the need for comprehensive health evaluations that extend beyond clinical indicators [9]. Consequently, HRQoL measures offer valuable insights into how diseases and their treatments affect patients’ daily lives, informing interventions that can improve health outcomes and quality of life [10].

Although HRQoL is recognized as a valuable outcome measure, there is a paucity of evidence regarding the utilization and implementation of HRQoL instruments within African healthcare research contexts [11]. Previous studies have highlighted challenges such as scarcity of culturally adapted and validated HRQoL instruments, disparities in research focus across different regions and populations, and inconsistencies in methodological approaches [12]. These gaps hinder the ability to compare results across studies, limit the generalizability of findings, and may result in the underrepresentation of certain populations or health conditions [13].

Understanding the current landscape of HRQoL research in Africa is essential for identifying areas of strength, uncovering gaps, and guiding future research efforts [14]. A comprehensive synthesis of existing studies can reveal patterns in the use of HRQoL instruments, the health conditions and populations studied, and the geographical distribution of research activities [15, 16]. Such information is crucial for healthcare providers, researchers, and policymakers aiming to implement patient-centered care approaches and improve health outcomes on the continent [16].

## Methods

This scoping review was conducted following the framework outlined by Arksey and O’Malley [17, 18], with methodological refinements informed by Levac et al. The process involved identifying the research questions, locating and selecting relevant studies, extracting and charting data, and summarizing and reporting the findings [17, 18]. The findings were reported in accordance with the Preferred Reporting Items for Systematic Reviews and Meta-Analyses Extension for Scoping Reviews (PRISMA-ScR) guidelines [19].

### Identifying the research question

The aim of this scoping review was to synthesize evidence on the use of health-related quality of life measures in Africa and provide a comprehensive overview of the current state of research.

The overarching research question was: What is known about the use of health-related quality of life measures in studies conducted among adult populations in Africa?

The specific research questions were:

i) How have health-related quality of life measures been used across diverse populations, health conditions, and geographical regions in Africa?
ii) What are the methodological characteristics of health-related quality of life studies conducted in Africa, including study designs, recruitment approaches, sample sizes, and data collection methods?
iii) Which generic and disease-specific health-related quality of life instruments have been used, and how have EQ-5D information-reporting practices been described, given its specific role in generating health-state utility values for health technology assessment and economic evaluation?

### Information sources and search strategy

A comprehensive systematic literature search was conducted to identify studies on the use of HRQoL instruments in Africa. The search strategy was developed by BMG, with assistance from the College of Health, Medicine and Wellbeing, the University of Newcastle librarian, to refine the search. The strategy was developed with a focus on identifying literature relevant to HRQoL in Africa using the PICOS framework recommended by the Joanna Briggs Institute (JBI) [20]. The search was conducted across five major electronic databases: Medline, Embase, Emcare, CINAHL, and Cochrane library. These databases were selected for their extensive coverage of health research literature and their relevance to the research scope. Search was conducted from database inception to August 2025. The search terms were adapted to account for variations in indexing systems and thesaurus terms across databases to enhance coverage and relevance. In addition to electronic database searches, manual hand searching was conducted to identify additional relevant studies from gray literature. This included searching the reference lists of included reviews, the first ten pages of Google Scholar, and the EuroQol database. The full search strategy is available in the online supplemental file 1 Table S1.

### Study eligibility and selection

Studies were eligible for inclusion if they used any health-related quality of life instrument to measure health outcomes in adult populations aged 18 years and older in African settings and were published in English. We considered observational and interventional studies, including randomized controlled trials, non-randomized trials, cohort studies, case-control studies, cross-sectional studies, mixed-methods studies, and descriptive studies. There were no restrictions on gender, ethnicity, or socioeconomic status. Studies were excluded if they involved pediatric populations without separate reporting for adults, were psychometric assessments, study protocols, reviews, reports, or commentaries, or if the full text could not be retrieved.

The studies identified through database and supplementary searches were exported into EndNote 20 reference management software, where duplicates were identified and removed. The remaining records were imported into Covidence for screening. Four reviewers (BMG, EAB, LGN, and JD) independently screened titles and abstracts using the predefined eligibility criteria. The same four reviewers then assessed the full texts of potentially eligible studies to determine final inclusion. Any conflicts or disagreements between reviewers were resolved through discussion.

### Data extraction and charting

A standardized data extraction form was developed using Microsoft Excel to systematically collect and organize relevant information from each included study. The data extraction form captured a range of information categorized into several key areas, including author details, year of publication, country, participant demographics, study design, sampling methods, health conditions studied, HRQoL instruments used, data collection modes, and information-reporting practices. It was pilot tested on a sample of ten studies to ensure clarity, consistency, and comprehensiveness. Four reviewers (BMG, EAB, LGN, and JD) independently extracted data from the included studies. Any discrepancies or disagreements between the reviewers were resolved through discussion.

### Synthesis of results

The extracted data were collated and summarized using a descriptive analysis approach to address the review’s objectives. Quantitative data was synthesized by calculating frequencies, percentages, median with interquartile range, and presented in tables and figures to illustrate patterns and distributions across studies. Visual representations, such as maps and charts, were created to depict the geographical distribution of studies, the health conditions examined, and the types of HRQoL instruments utilized.

The findings were organized narratively according to the main areas of interest: methodological characteristics of the studies, geographical distribution, populations and health conditions studied, and types of HRQoL instruments used.

## Results

### Study selection

**Fig. 1:**
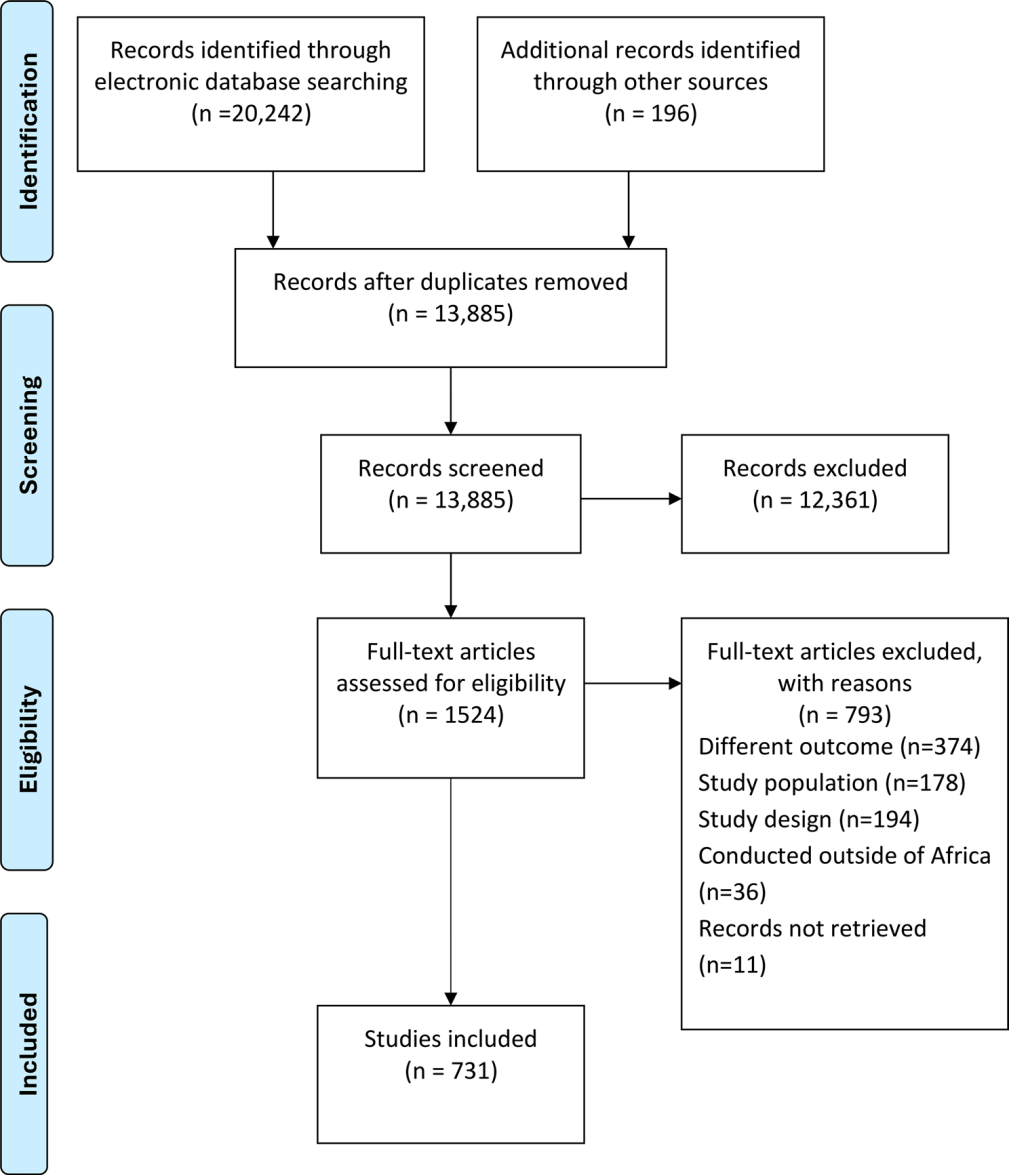
PRISMA flow diagram depicting the study selection process

A total of 20,242 records were identified through electronic database searches, and an additional 196 records were located through other sources. After the removal of duplicates (*n* = 6,553), 13,885 unique records remained for screening. The titles and abstracts of these records were assessed for relevance, resulting in the exclusion of 12,361 records that did not meet the inclusion criteria. Consequently, 1,524 articles were retrieved and evaluated for eligibility based on the predefined criteria. During the full-text assessment, 793 articles were excluded for the following reasons: different outcome measures (*n* = 374), study population (participants aged ≤ 18 years) (*n* = 178), study design (psychometric assessments, study protocols, reviews, reports, or commentaries (*n* = 194), conducted outside of Africa (*n* = 36), and records not retrieved (*n* = 11). In the end, 731 studies met all eligibility criteria and were included in the final analysis (Online supplemental file 2). The study selection process is illustrated in the flow diagram presented in Fig. 1.

### Study characteristics

#### Publication trend and study design

Studies on HRQoL in Africa began to emerge in the late 1990s, with a notable increase in publication activity observed since 2008, during which 65.8% (*n* = 481) of the included studies were published (Online supplemental file 1 Figure S1). Of the 731 studies included in this review, the majority (74.2%, *n* = 542) employed descriptive study designs, reflecting a primary focus on exploring and characterizing HRQoL outcomes in Africa. Observational studies accounted for 18.7% (*n* = 137), while randomized controlled trials (RCTs) constituted 5.1% (*n* = 37). Quasi-experimental studies and mixed-methods designs were less frequently utilized, representing 1.4% (*n* = 10) and 0.7% (*n* = 5) of the studies, respectively (Table 1).

#### Sample size and data collection method

The included studies varied in terms of methodological characteristics, including sample size, sampling methods, and modes of data collection. The sample size ranged widely, with a median of 210 participants (Q1-Q3: 101–394; interquartile range (IQR): 293). Among the sampling methods, convenience sampling was the most frequently employed, used in 24.9% (*n* = 182) of studies, followed by consecutive sampling in 20.8% (*n* = 152). In contrast, less commonly used methods included cluster sampling (1.8%, *n* = 13) and multistage sampling (2.5%, *n* = 18). Notably, 15.6% (*n* = 114) of studies did not specify their sampling method, highlighting a gap in methodological reporting (Table 1).

Data were mainly collected using interviewer-administered methods in 75.8% (*n* = 554) of studies. Self-administered questionnaires were utilized in 16.3% (*n* = 119) of the studies, providing participants with the autonomy to complete the surveys independently. A mixed approach combining both interviewer-administered and self-administered methods was employed in 6.3% (*n* = 46) of studies. A small proportion of studies (1.6%, *n* = 12) did not report their data collection methods.

#### Study setting

Most studies (70.3%, *n* = 514) were conducted in hospital settings. Other healthcare settings included health centers or clinics (8.1%, *n* = 59), specialty clinics (2.6%, *n* = 19), and care centers (1.0%, *n* = 7). Non-healthcare settings accounted for 13.6% of studies and included community settings (12.6%, *n* = 92) and educational settings, such as universities and schools (1.0%, *n* = 7). A smaller proportion of studies (1.4%, *n* = 10) were conducted in mixed settings that integrated community and healthcare facilities (Table 1).

**Table 1:** Summary of included study characteristics (*n* = 731)

Characteristics	Frequency	Percentage (%)
**Study design type**		
Descriptive studies	542	74.2
Observational	137	18.7
Randomized controlled trial	37	5.1
Quasi-experimental studies	10	1.4
Mixed methods research	5	0.7
**Participant type**		
Patients	616	84.3
General population	60	8.2
Combined patients and general population	7	1.0
Geriatric population (elderly)	18	2.5
Mothers or caregivers of children	27	3.7
Individuals with disabilities	3	0.4
**Study setting**		
Hospital	514	70.3
Community setting	92	12.6
Clinic or health center	59	8.1
Specialty clinic	19	2.6
Community/health facility	10	1.4
Care center	7	1.0
University/school	7	1.0
Other*	15	2.1
Not reported	8	1.1
**Sampling method**		
***Probability sampling***:		
Simple Random	107	14.6
Systematic random sampling	49	6.7
Stratified Sampling	31	4.2
Cluster sampling	13	1.8
Multistage sampling	18	2.5
***Non-probability sampling***:		
Purposive sampling	59	8.1
Consecutive sampling	152	20.8
Convenience sampling	182	24.9
Other	6	0.8
Not reported	114	15.6
**Data collection modes**		
Interviewer-administered	554	75.8
Self-reported	119	16.3
Mixed approach	46	6.3
Not reported	12	1.6
**Sample size**		
< 101 (Below 25th percentile)	188	25.7
101–210 (25th to 50th percentile)	181	24.8
211–394 (50th to 75th percentile)	187	25.6
> 394 (Above 75th percentile)	175	23.9
Median (Q1–Q3; IQR)	210 (101–394; 293)	

^*^ Unspecified rural settings, factory, government, and non-government workplaces

IQR: interquartile range

### Geographical distribution and frequency of HRQoL studies

**Fig. 2:**
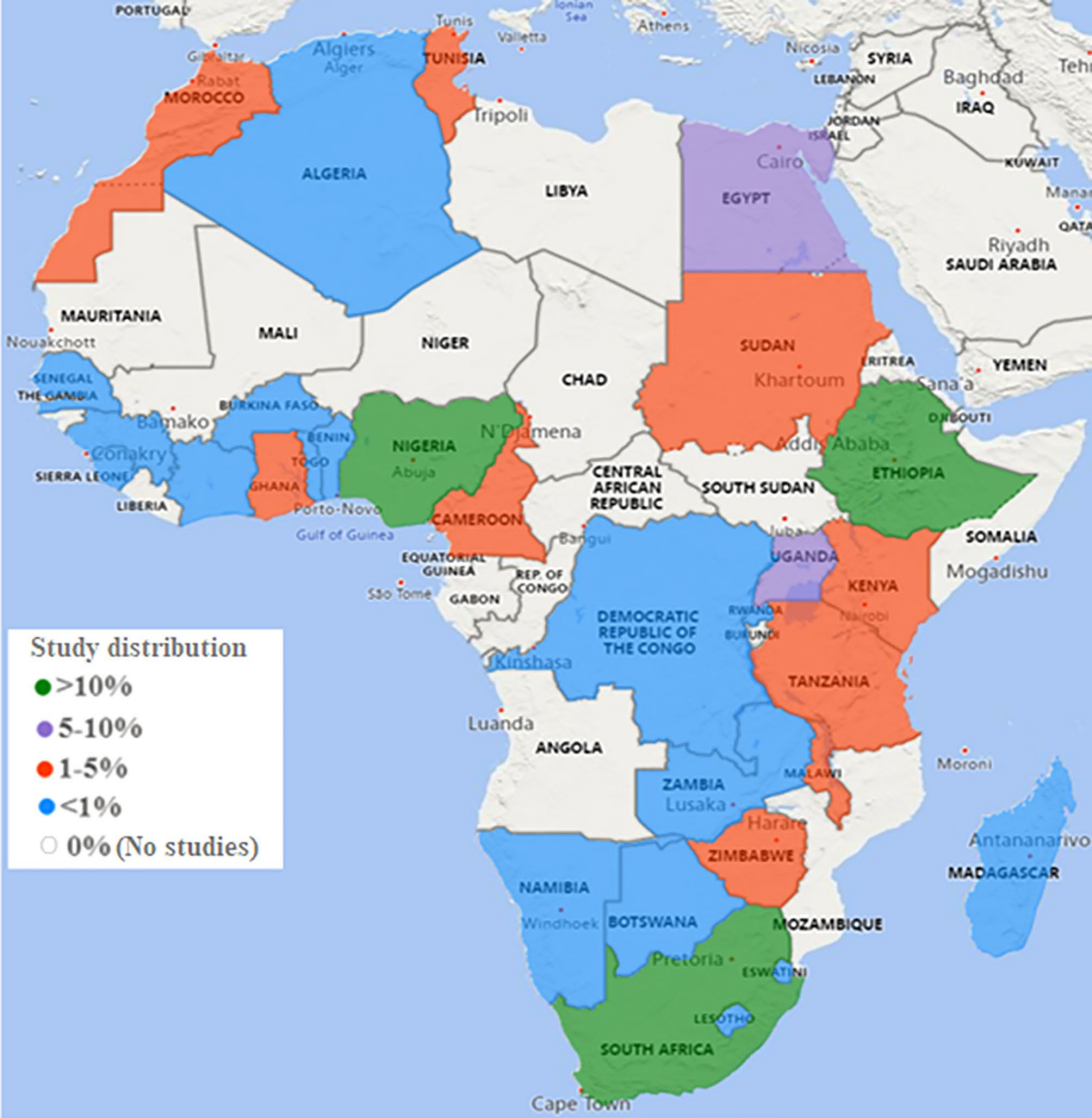
The geographic distribution of HRQoL study coverage across African countries is represented by a color-coded gradient. Countries contributing more than 10% of the total studies are highlighted in green, indicating high research activity. Contributions ranging from 5% to 10% are shown in purple, while countries contributing between 1% and 5% are marked in red. Countries with less than 1% contribution are shaded in blue, and those with no reported HRQoL studies are displayed in white, except for Mozambique, which is included in a study conducted across multiple countries. Map generated using Microsoft Power BI

Figure 2 shows the geographical distribution of HRQoL studies across African countries, using a color-coded gradient to represent varying levels of research activity. Most of the studies (95.5%, *n* = 698) were conducted in 31 African countries. However, more than half (51%, *n* = 373) of the studies were conducted in three countries, i.e., Nigeria (21.8%, *n* = 159), Ethiopia (14.8%, *n* = 108), and South Africa (14.5%, *n* = 106). In contrast, several countries, including Chad, the Central African Republic, and Eritrea (marked in white), had no reported HRQoL studies (Online supplemental file 1 Table S2).

### Target populations and common health conditions

Most studies (84.8%, *n* = 620) included participants of both sexes. Regarding target populations, most studies (84.3%, *n* = 616) investigated patient populations, with only 8.2% (*n* = 60) focusing on healthy or general populations. Smaller proportions of studies targeted specific groups such as mothers or caregivers of children (3.7%, *n* = 27), elderly or geriatric individuals (2.5%, *n* = 18), and disabled groups (0.4%, *n* = 3 (Table 1).

**Fig. 3:**
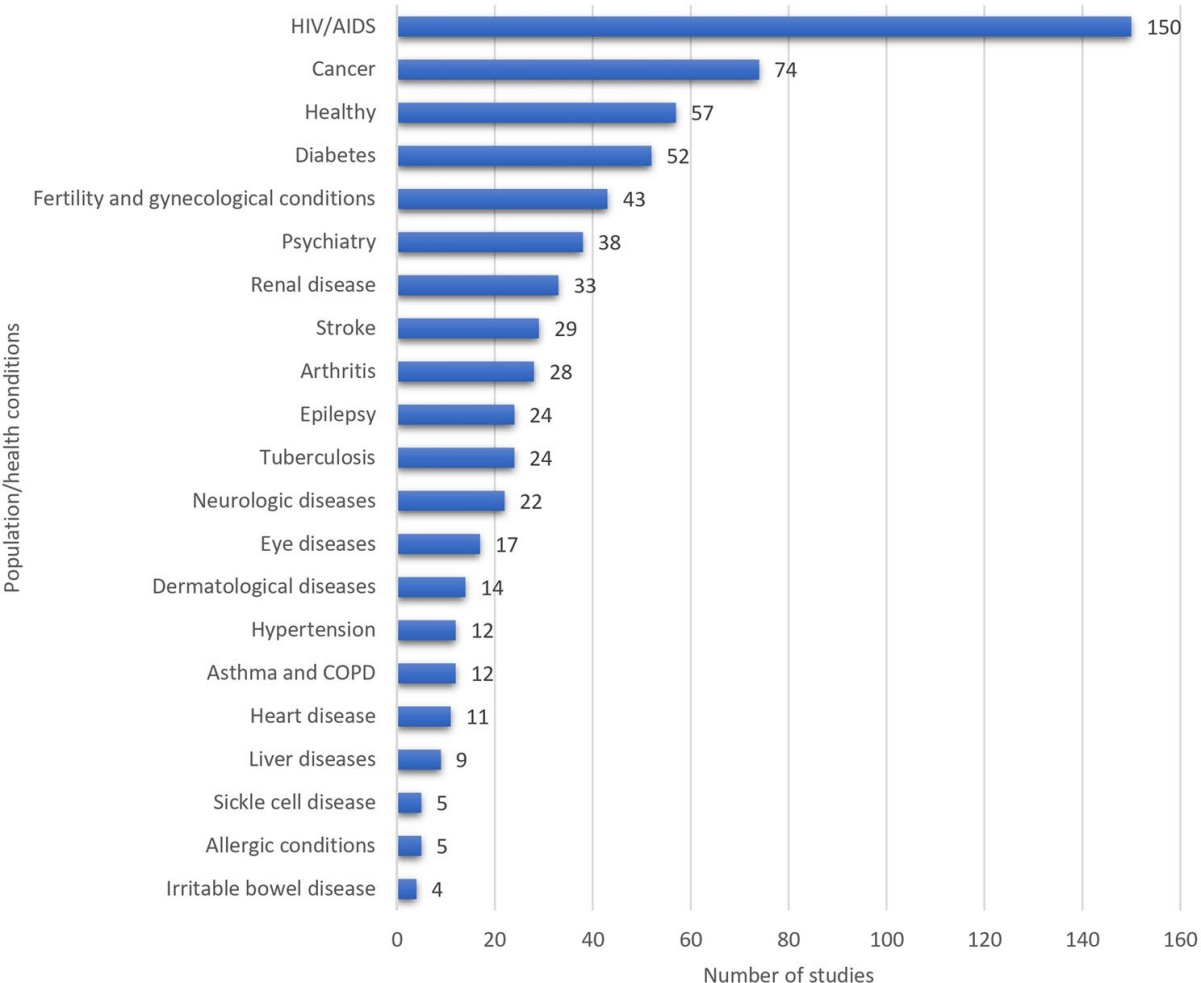
Distribution of study populations and common health conditions examined using HRQoL instruments

The distribution of health conditions examined using HRQoL instruments is presented in Fig. 3. The most commonly studied conditions included HIV/AIDS (20.5%, *n* = 150), cancer (10.1%, *n* = 74), diabetes (7.1%, *n* = 52), fertility and gynecological conditions (5.9%, *n* = 43), psychiatric disorders (5.2%, *n* = 38), and renal diseases (4.5%, *n* = 33).

### Types of HRQoL instruments utilized in Africa

**Fig. 4:**
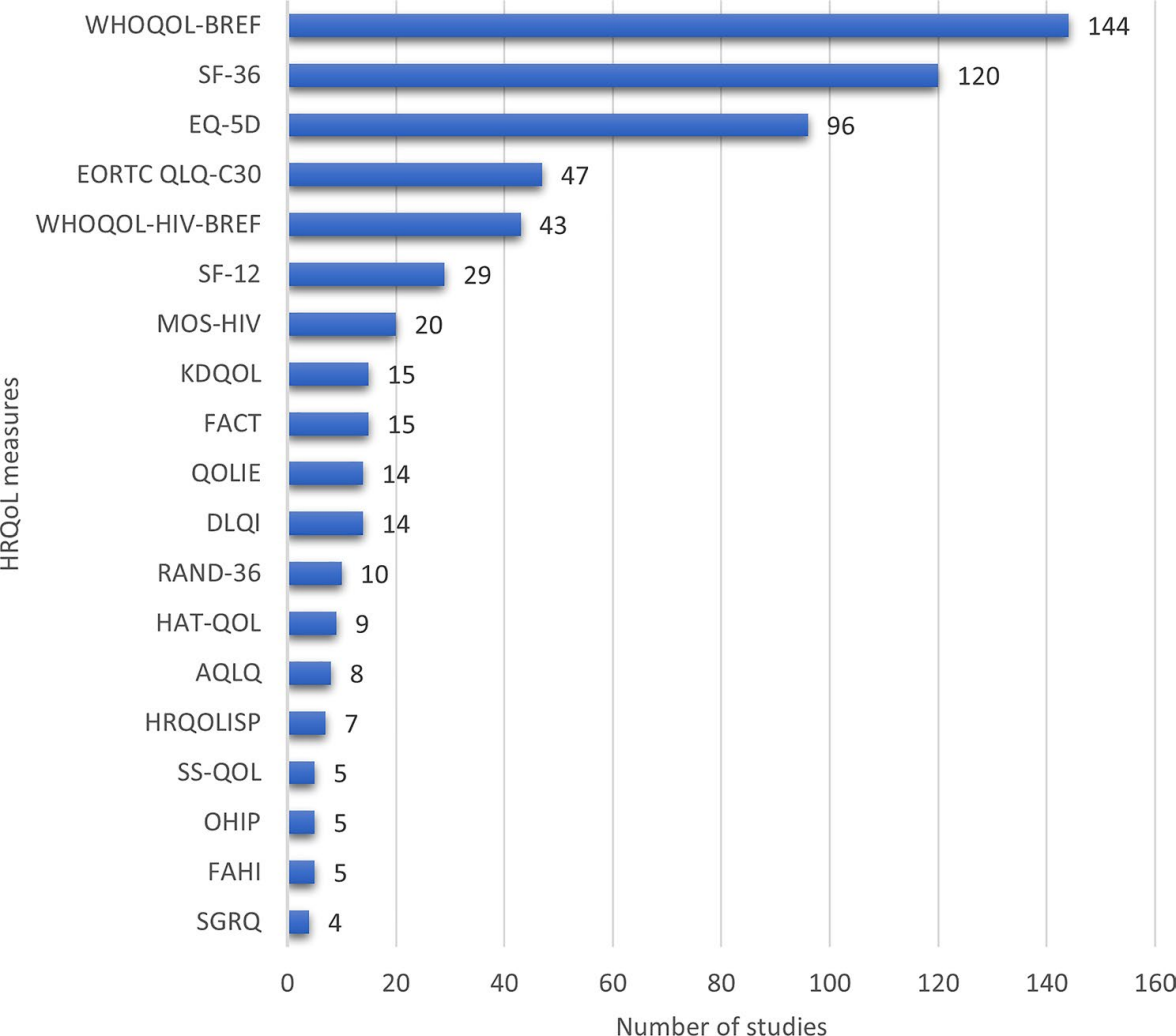
Distribution of commonly used HRQoL measures in Africa. SF-12: 12-Item Short Form Survey, SF-36: 36-Item Short Form Survey, AQLQ: Asthma Quality of Life Questionnaire, DLQI: Dermatology Life Quality Index, EORTC QLQ-C30: European Organization for Research and Treatment of Cancer Quality of Life Questionnaire-Core 30, EQ-5D: EuroQol 5 Dimensions, FACT: Functional Assessment of Cancer Therapy, FAHI: Functional Assessment of HIV Infection, HRQOLISP: Health related quality of life in stroke patients, HAT-QOL: HIV/AIDS-Targeted Quality of Life Instrument, KDQOL: Kidney Disease Quality of Life Instrument, MOS-HIV: Medical Outcome Study-HIV Health Survey, OHIP: Oral Health Impact Profile, QOLIE: Quality of Life in Epilepsy, SGRQ: St. George’s Respiratory Questionnaire, SS-QOL: Stroke-Specific Quality of Life Questionnaire: WHOQOL-BREF: World Health Organization Quality of Life Brief Version, WHOQOL-HIV-BREF: World Health Organization Quality of Life Instrument for People Living with HIV, Short Version

As shown in Fig. 4, a wide range of HRQoL instruments have been used in studies across Africa. The most commonly used instruments were the World Health Organization Quality of Life: Brief Version (WHOQOL-BREF): 19.7% (*n* = 144), the 36-Item Short Form Survey (SF-36): 16.4% (*n* = 120), and the EQ-5D: 13.1% (*n* = 96). Other commonly used instruments included the WHOQOL-HIV-BREF (5.9%, *n* = 43), the European Organization for Research and Treatment of Cancer Quality of Life Questionnaire (EORTC QLQ, 6.4%, *n* = 47), and the 12-Item Short Form Survey (SF-12, 4.0%, *n* = 29).

### Use of EQ-5D versions and information-reporting practices

**Fig. 5:**
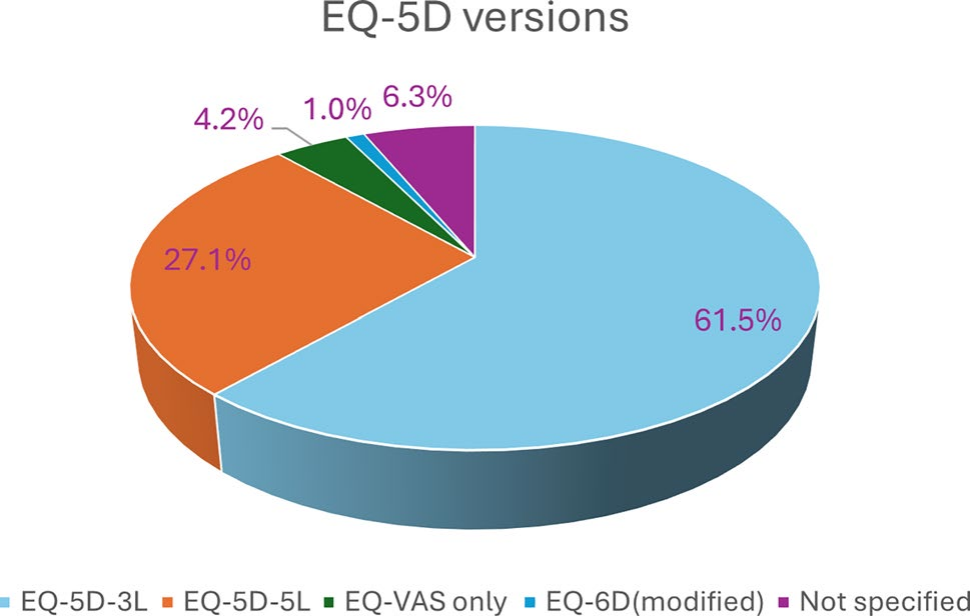
Distribution of EQ-5D versions utilized in HRQoL studies in Africa (*n* = 96)

Among the 96 studies that used the EQ-5D, the EQ-5D-3L was the most commonly used version, reported in 61.5% (*n* = 59) of these studies. The EQ-5D-5L was employed in 27.1% (*n* = 26), while a small proportion (4.2%, *n* = 4) used the EQ-VAS alone, without the accompanying descriptive system. Additionally, 6.3% (*n* = 6) of the studies did not specify which version of the EQ-5D was used (Fig. 5).

In studies utilizing the EQ-5D-3L (*n* = 59), information-reporting practices showed considerable variation. Comprehensive reporting, which included the descriptive profile, index score (derived from a value set), and EQ-VAS, was observed in 20.3% (*n* = 12) of the studies. Partial reporting was also common: 22.0% (*n* = 13) included the descriptive profile and EQ-VAS, 10.2% (*n* = 6) included the descriptive profile and index value, and another 10.2% (*n* = 6) reported index score and EQ-VAS. Single-component reporting was less frequent, with 13.5% (*n* = 8) reporting only the descriptive profile, 14.5% (*n* = 8) reporting only the index score, and another 10.2% (*n* = 6) reporting only the EQ-VAS score (Online supplemental file 1 Table S3).

For studies using EQ-5D-5L (*n* = 26), the majority (65.4%, *n* = 17) included all three components: descriptive profile, index score, and EQ-VAS. A few studies (11.5%, *n* = 3) reported both the descriptive profile and EQ-VAS, while an equal proportion (11.5%, *n* = 3) reported the index score and EQ-VAS, and another (7.7%, *n* = 2) reported the descriptive profile and index scores. Only one (3.8%) study the descriptive profile alone (Online supplemental file 1 Table S3).

## Discussion

This scoping review provided a comprehensive overview of the use of HRQoL measures in Africa, revealing significant insights into the current landscape of patient-centered research across the continent. The majority of the studies were concentrated in a few countries, while several others remain underrepresented. Most studies employed descriptive designs, focused on hospital settings, and investigated conditions such as HIV/AIDS, cancer, and diabetes. Generic HRQoL instruments, including WHOQOL-BREF, SF-36, and EQ-5D, were commonly used, though EQ-5D reporting practices were inconsistent. The findings highlighted both progress and persistent gaps in the application of HRQoL instruments, offering valuable implications for future research and healthcare policy.

A greater number of HRQoL studies have been conducted since 2008. This rise may be due to several overlapping developments. First, the World Health Organization’s 2008–2013 Action Plan for the Global Strategy for the Prevention and Control of Noncommunicable Diseases raised global awareness of chronic disease prevention and management, especially in low- and middle-income countries, urging stakeholders to urgently address the increasing burden of noncommunicable diseases. Second, the expansion of antiretroviral therapy programs in Africa shifted HIV care from solely survival towards enhancing long-term functioning and quality of life for people living with HIV [21]. Finally, this period coincided with increased investment in African health research capacity, including initiatives like the Initiative to Strengthen Health Research Capacity in Africa, the Consortium for Advanced Research Training in Africa, and the Wellcome Trust African Institutions Initiative [22–24]. However, since this review mapped publication trends rather than directly exploring their causes, these explanations should be viewed as contextual rather than definitive.

The geographical distribution of HRQoL studies across Africa demonstrated disparity, with over half of the included studies conducted in just three countries: Nigeria, Ethiopia, and South Africa. This was in line with the findings from previous studies of health research in Africa, highlighting the persistent inequality in research capacity and funding across the continent [25–27]. The lack of HRQoL studies in about a third of countries on the African continent underscores the urgent need for targeted research initiatives in underrepresented regions [26]. This disparity not only limits our understanding of health outcomes in these areas but also raises concerns about health equity and the generalizability of existing HRQoL data to diverse African populations [25, 27]. Addressing this inequality requires concerted efforts to build research capacity, establish collaborative networks, and prioritize funding for HRQoL studies in underrepresented countries [25–27].

The predominance of descriptive studies (74.2%) highlights a strong focus on exploratory research, consistent with the relatively recent adoption of HRQoL measures in many African countries [28]. Although valuable for establishing baseline information, this approach underscores the pressing need for interventional studies, such as RCTs, to evaluate the impact of health interventions on HRQoL outcomes [29]. Additionally, the widespread use of interviewer-administered data collection methods demonstrates efforts to overcome literacy barriers and ensure data quality, which is particularly critical in Africa’s diverse linguistic and cultural contexts [28, 30]. However, this resource-intensive approach raises concerns regarding potential interviewer bias, highlighting the importance of culturally sensitive training for research personnel [28, 31]. Furthermore, the significant variation in sample sizes and sampling methods across studies shows the diversity of research approaches, but also highlights inconsistencies that may limit the comparability and generalizability of findings [32, 33].

A wide range of HRQoL instruments were utilized, with the WHOQOL-BREF, SF-36, and EQ-5D being the most commonly used. The preference for these generic HRQoL instruments may be because of their widespread acceptance, ease of use, and availability of validated versions in various languages [28]. The use of disease-specific instruments for conditions such as HIV/AIDS, cancer, and kidney disease indicates the importance of targeted assessments, emphasizing the need to consider their applicability in diverse African contexts. Moreover, the use of EQ-5D instruments was reported in many studies, with the EQ-5D-3 L version being more common than the EQ-5D-5 L. Inconsistencies were observed in the information reporting practices of the EQ-5D data, with some studies providing comprehensive information (descriptive profile, index score, and EQ-VAS), and others reporting only partial data. Some studies did not specify the instrument version or reported only selected components of EQ-5D data, limiting the utility of their findings and highlighting the need for standardized reporting guidelines to enhance the utility and comparability of HRQoL data [34].

### Limitations

While this scoping review provides valuable insights into the use of HRQoL measures in Africa, several limitations should be acknowledged. First, the review was restricted to studies published in English, which may have excluded relevant research published in other languages. This may have led to an underrepresentation of HRQoL research from certain regions. Although five electronic databases and supplementary sources, including Google Scholar, EuroQol, and reference lists, were searched, dedicated grey literature databases such as OpenGrey were not searched. Second, the review focused on adult populations, excluding studies involving participants under 18 years old. This limits our understanding of HRQoL measures in pediatric populations across Africa. Finally, the heterogeneity in study designs, sample sizes, and reporting practices made it challenging to draw definitive conclusions. These limitations should be considered when interpreting the results.

### Implications

The findings of this scoping review have important implications for policy, practice, and research in Africa. From a policy perspective, the concentration of HRQoL studies in a limited number of countries highlights the need for more geographically inclusive investment in health outcomes research. National and regional research agendas should prioritise underrepresented countries and explicitly include vulnerable populations, such as persons with disabilities, displaced individuals, rural communities, and socioeconomically disadvantaged groups. Greater inclusion of these groups is essential to ensure that HRQoL evidence reflects the diversity of African populations and can inform equitable health policy and resource allocation.

From a practice perspective, the dominance of hospital-based studies highlights the need to expand HRQoL assessment into primary care, community, and humanitarian settings. This would help capture the experiences of people who face barriers to facility-based care, including those affected by disability, displacement, poverty, or geographic isolation. Routine use of culturally appropriate HRQoL measures could support patient-centred care, identify unmet needs, and guide service planning in resource-constrained settings.

From a research perspective, inconsistencies in HRQoL reporting, especially for EQ-5D instruments, highlight the need for clearer and more standardised reporting practices. Future studies should move beyond descriptive designs by employing longitudinal and interventional methods to explore how health interventions affect HRQoL over time. Further research should also focus on vulnerable and underrepresented populations, and evaluate the translation, cultural adaptation, and measurement properties of commonly used HRQoL instruments across diverse African contexts. Addressing these gaps would enhance the quality, comparability, equity, and policy relevance of HRQoL evidence across African health systems.

## Conclusions

This scoping review highlighted the growing integration of HRQoL measures in African health research, reflecting progress in addressing the continent’s unique health challenges. However, significant gaps remain, including limited geographical diversity, inconsistencies in reporting practices, and methodological weaknesses. Future research should prioritize expanding studies to underrepresented regions, adopting standardized reporting guidelines, and enhancing methodological rigor to improve the quality and comparability of findings. Addressing these gaps is essential to generate robust, contextually relevant evidence that can inform health policy, support equitable resource allocation, and improve patient-centered care across Africa. 

## Supplementary Information

Below is the link to the electronic supplementary material.

Supplementary Material 1

Supplementary Material 2

## Data Availability

All data generated or analysed during this study are included in this published article and its supplementary information files.
