# Supplementary material for: Mapping health-related quality of life measurement tools in Africa: a scoping review
Source: J Patient Rep Outcomes. 2026 May 30;10:135. doi: 10.1186/s41687-026-01100-6 (PMC13442791; doi:10.1186/s41687-026-01100-6)
Supplement: Supplementary file 1 [file 41687_2026_1100_MOESM1_ESM.docx]

**Supplementary file 1**

**Table S1:** Search Strategy for electronic databases

| **Ovid MEDLINE(R) search strategy** | | | | |
| --- | --- | --- | --- | --- |
| 1. | | | "Quality of Life"/ | |
| 2. | | | (Health adj related adj quality of life). ab,hw,kw,ti. | |
| 3. | | | (Health adj profile$). ab, hw,kw,ti. | |
| 4. | | | "activities of daily living"/ or functional status/ | |
| 5. | | | Pain measurement/ or visual analog scale/ | |
| 6. | | | patient-reported outcome measures/ or patient health questionnaire/ or self-report/ | |
| 7. | | | (Preference-based adj health adj1 measure$).ab,hw,kw,ti. | |
| 8. | | | (Generic adj health profile measure$).ab,hw,kw,ti. | |
| 9. | | | (Disease adj1 specific adj health status measure$).ab,hw,kw,ti. | |
| 10. | | | (Health adj technology adj assessment$).ab,hw,kw,ti. | |
| 11. | | | (Psychometr$ adj1 measurement$).ab,hw,kw,ti. | |
| 12. | | | (Health adj1 valuation$).ab,hw,kw,ti. | |
| 13. | | | EuroQol$.ab,hw,kw,ti. | |
| 14. | | | EQ?5D.ab,hw,kw,ti. | |
| 15. | | | EQ?5D?5L.ab,hw,kw,ti. | |
| 16. | | | EQ?5D?3L.ab,hw,kw,ti. | |
| 17. | | | EQ?5D?Y.ab,hw,kw,ti. | |
| 18. | | | EQ?5D?Y?5L.ab,hw,kw,ti. | |
| 19. | | | EQ?5D?Y?3L.ab,hw,kw,ti. | |
| 20. | | | (EQ?5D adj index).ab,hw,kw,ti. | |
| 21. | | | Short?Form 36.ab,hw,kw,ti. | |
| 22. | | | Short?Form 6D.ab,hw,kw,ti. | |
| 23. | | | (Health adj Utilit$ adj Index).ab,hw,kw,ti. | |
| 24. | | | (Quality adj1 Well?Being).ab,hw,kw,ti. | |
| 25. | | | (P?ediatric adj Quality of Life adj Questionnaire).ab,hw,kw,ti. | |
| 26. | | | (Quality of Life adj Profile).ab,hw,kw,ti. | |
| 27. | | | (Perceived adj Illness adj Experience).ab,hw,kw,ti. | |
| 28. | | | (Generic adj Health adj Questionnaire).ab,hw,kw,ti. | |
| 29. | | | (Child$ adj Health adj1 Illness adj Profile).ab,hw,kw,ti. | |
| 30. | | | (Child$ adj Health adj Questionnaire).ab,hw,kw,ti. | |
| 31. | | | (Child adj Quality of Life adj Questionnaire).ab,hw,kw,ti. | |
| 32. | | | HRQoL.ab,kf,kw,ti. | |
| 33. | | | QoL.ab,kf,kw,ti. | |
| 34. | | | Health status index.ab,kf,kw,ti. | |
| 35. | | | Health status utilit$.ab,kf,kw,ti. | |
| 36. | | | SF?36.ab,kf,kw,ti. | |
| 37. | | | SF?6D.ab,kf,kw,ti. | |
| 38. | | | HUI.ab,kf,kw,ti. | |
| 39. | | | QWB.ab,kf,kw,ti. | |
| 40. | | | Africa, Western/ or Africa, Central/ or "Africa South of the Sahara"/ or Africa/ or Africa, Northern/ or Africa, Eastern/ or Africa, Southern/ | |
| 41. | | | Algeria/ | |
| 42. | | | Angola/ | |
| 43. | | | Benin/ | |
| 44. | | | Botswana/ | |
| 45. | | | Burkina Faso/ | |
| 46. | | | Burundi/ | |
| 47. | | | Cabo Verde/ | |
| 48. | | | Cameroon/ | |
| 49. | | | Central African Republic/ | |
| 50. | | | Chad/ | |
| 51. | | | Comoros/ | |
| 52. | | | "Democratic Republic of the Congo"/ | |
| 53. | | | Congo/ | |
| 54. | | | Cote d'Ivoire/ | |
| 55. | | | Djibouti/ | |
| 56. | | | Egypt/ | |
| 57. | | | Equatorial Guinea/ | |
| 58. | | | Eritrea/ | |
| 59. | | | Eswatini/ | |
| 60. | | | Ethiopia/ | |
| 61. | | | Gabon/ | |
| 62. | | | Gambia/ | |
| 63. | | | Ghana/ | |
| 64. | | | Guinea/ | |
| 65. | | | Guinea-Bissau/ | |
| 66. | | | Kenya/ | |
| 67. | | | Lesotho/ | |
| 68. | | | Liberia/ | |
| 69. | | | Libya/ | |
| 70. | | | Madagascar/ | |
| 71. | | | Malawi/ | |
| 72. | | | Mali/ | |
| 73. | | | Mauritania/ | |
| 74. | | | Mauritius/ | |
| 75. | | | Morocco/ | |
| 76. | | | Mozambique/ | |
| 77. | | | Namibia/ | |
| 78. | | | Niger/ | |
| 79. | | | Nigeria/ | |
| 80. | | | Rwanda/ | |
| 81. | | | (Sao Tome and Principe).ab,hw,kw,ti. | |
| 82. | | | Senegal/ | |
| 83. | | | Seychelles/ | |
| 84. | | | Sierra Leone/ | |
| 85. | | | Somalia/ | |
| 86. | | | South Africa/ | |
| 87. | | | South Sudan/ | |
| 88. | | | Sudan/ | |
| 89. | | | Tanzania/ | |
| 90. | | | Togo/ | |
| 91. | | | Tunisia/ | |
| 92. | | | Uganda/ | |
| 93. | | | Zambia/ | |
| 94. | | | Zimbabwe/ | |
| 95. | | | 1 or 2 or 3 or 4 or 5 or 6 or 7 or 8 or 9 or 10 or 11 or 12 or 13 or 14 or 15 or 16 or 17 or 18 or 19 or 20 or 21 or 22 or 23 or 24 or 25 or 26 or 27 or 28 or 29 or 30 or 31 or 32 or 33 or 34 or 35 or 36 or 37 or 38 or 39 | |
| 96. | | | 40 or 41 or 42 or 43 or 44 or 45 or 46 or 47 or 48 or 49 or 50 or 51 or 52 or 53 or 54 or 55 or 56 or 57 or 58 or 59 or 60 or 61 or 62 or 63 or 64 or 65 or 66 or 67 or 68 or 69 or 70 or 71 or 72 or 73 or 74 or 75 or 76 or 77 or 78 or 79 or 80 or 81 or 82 or 83 or 84 or 85 or 86 or 87 or 88 or 89 or 90 or 91 or 92 or 93 or 94 | |
| 97. | | | 95 and 96 | |
| **Note:** Comparable searches were applied in Embase and Emcare, with database-specific subject headings and syntax adapted as required. | | | | |
| **EBSCOhost/CINAHL search strategy** | | | | |
| **Search line** | | **Concept** | | **Search syntax** |
| S1 | | Africa/geographic subject headings | | (MH "Africa") OR (MH "Africa South of the Sahara") OR (MH "Africa, Western") OR (MH "Africa, Central") OR (MH "Africa, Southern") OR (MH "Africa, Northern") OR (MH "Africa, Eastern") |
| S2 | | Geographic term: Algeria | | "Algeria" |
| S3 | | Geographic term: Angola | | "Angola" |
| S4 | | Geographic term: Benin | | (MH "Benin") |
| S5 | | Geographic term: Botswana | | (MH "Botswana") OR "botswana" |
| S6 | | Geographic term: Burkina Faso | | "burkina faso" |
| S7 | | Geographic term: Burundi | | (MH "Burundi") OR "Burundi" |
| S8 | | Geographic term: Cabo Verde | | "Cabo verde" |
| S9 | | Geographic term: Cameroon | | "Cameroon" |
| S10 | | Geographic term: Central African Republic | | (MH "Central African Republic") OR "central african republic" |
| S11 | | Geographic term: Chad | | (MH "Chad") OR "Chad" |
| S12 | | Geographic term: Comoros | | "comoros" |
| S13 | | Geographic term: Democratic Republic of the Congo | | (MH "Democratic Republic of the Congo") OR "democratic republic of congo" |
| S14 | | Geographic term: Congo | | "Congo" |
| S15 | | Geographic term: Cote d'Ivoire | | (MH "Cote d'Ivoire") |
| S16 | | Geographic term: Djibouti | | (MH "Djibouti") |
| S17 | | Geographic term: Egypt | | (MH "Egypt") OR "Egypt" |
| S18 | | Geographic term: Equatorial Guinea | | (MH "Equatorial Guinea") OR "equatorial guinea" |
| S19 | | Geographic term: Eritrea | | (MH "Eritrea") OR "eritrea" |
| S20 | | Geographic term: Eswatini/Swaziland | | "eswatini or swaziland" |
| S21 | | Geographic term: Ethiopia | | "ethiopia" |
| S22 | | Geographic term: Gabon | | "Gabon" |
| S23 | | Geographic term: Gambia | | "Gambia" |
| S24 | | Geographic term: Ghana | | (MH "Ghana") |
| S25 | | Geographic term: Guinea | | (MH "Guinea") OR "Guinea" |
| S26 | | Geographic term: Guinea-Bissau | | (MH "Guinea-Bissau") OR "Guinea-Bissau" |
| S27 | | Geographic term: Kenya | | (MH "Kenya") |
| S28 | | Geographic term: Lesotho | | (MH "Lesotho") OR "Lesotho" |
| S29 | | Geographic term: Liberia | | (MH "Liberia") |
| S30 | | Geographic term: Libya | | "Libya" |
| S31 | | Geographic term: Madagascar | | (MH "Madagascar") OR "Madagascar" |
| S32 | | Geographic term: Malawi | | (MH "Malawi") OR "Malawi" |
| S33 | | Geographic term: Mali | | (MH "Mali") |
| S34 | | Geographic term: Mauritania | | (MH "Mauritania") OR "Mauritania" |
| S35 | | Geographic term: Mauritius | | "Mauritius" |
| S36 | | Geographic term: Morocco | | (MH "Morocco") OR "Morocco" |
| S37 | | Geographic term: Mozambique | | (MH "Mozambique") OR "Mozambique" |
| S38 | | Geographic term: Namibia | | (MH "Namibia") OR "Namibia" |
| S39 | | Geographic term: Niger | | (MH "Niger") OR "Niger" |
| S40 | | Geographic term: Nigeria | | (MH "Nigeria") |
| S41 | | Geographic term: Rwanda | | (MH "Rwanda") |
| S42 | | Geographic term: Sao Tome and Principe | | "sao tome and principe" |
| S43 | | Geographic term: Senegal | | (MH "Senegal") |
| S44 | | Geographic term: Seychelles | | "Seychelles" |
| S45 | | Geographic term: Sierra Leone | | (MH "Sierra Leone") |
| S46 | | Geographic term: Somalia | | (MH "Somalia") OR "Somalia" |
| S47 | | Geographic term: South Africa | | (MH "South Africa") OR "South Africa" |
| S48 | | Geographic term: South Sudan | | "South Sudan" |
| S49 | | Geographic term: Sudan | | (MH "Sudan") OR "Sudan" |
| S50 | | Geographic term: Tanzania | | (MH "Tanzania") |
| S51 | | Geographic term: Togo | | (MH "Togo") |
| S52 | | Geographic term: Tunisia | | (MH "Tunisia") OR "Tunisia" |
| S53 | | Geographic term: Uganda | | (MH "Uganda") OR "Uganda" |
| S54 | | Geographic term: Zambia | | (MH "Zambia") |
| S55 | | Geographic term: Zimbabwe | | (MH "Zimbabwe") OR "Zimbabwe" |
| S56 | | Quality of life subject headings | | (MH "Quality of Life") OR (MH "Health and Life Quality (Iowa NOC)") OR (MH "Psychological Well-Being") |
| S57 | | HRQoL/QoL keywords | | health related quality of life or hrqol or quality of life or qol |
| S58 | | Pain measurement terms | | (MH "Pain Measurement") OR "pain measurement or pain assessment or pain scale" |
| S59 | | Outcome assessment/PROM terms | | (MH "Outcome Assessment") OR (MH "Patient-Reported Outcomes") OR (MH "Outcomes (Health Care)") OR (MH "Patient Assessment") OR (MH "Outcome Assessment Information Set") OR "patient reported outcome measures/ or patient outcome assessment/" |
| S60 | | Preference-based measure term | | "preference based measure/assessment" |
| S61 | | Preference-based measure term | | "preference based measure/assessment" |
| S62 | | Generic health profile measure term | | "generic health profile measure" |
| S63 | | Health status measure terms | | (MH "Health Status") OR "disease health status measure" |
| S64 | | Psychometric measure term | | "psychometric measures" |
| S65 | | EuroQoL term | | "EuroQoL" |
| S66 | | European quality of life questionnaire term | | "european quality of life questionnaire" |
| S67 | | EQ-5D term | | "EQ-5D" |
| S68 | | EQ-5D-5L exact phrase | | "eq-5d-5l" |
| S69 | | EQ-5D-5L keyword | | eq-5d-5l |
| S70 | | EQ-5D-Y keyword | | eq-5d-y |
| S71 | | EQ-5D-Y-5L keyword | | EQ-5D-Y-5L |
| S72 | | EQ-5D-Y-3L keyword | | EQ-5D-Y-3L |
| S73 | | SF-36 terms | | (MH "Short Form-36 Health Survey (SF-36)") OR "short form 36 or sf-36" |
| S74 | | EuroQol 5-Dimension term | | EuroQol 5-Dimension |
| S75 | | Functional assessment/FACT term | | (MH "Functional Assessment") OR "functional assessment of cancer therapy" |
| S76 | | QLQ-C30 term | | "QLQ-C30 (Quality of Life Questionnaire-Core 30)" |
| S77 | | PROMIS term | | PROMIS (Patient-Reported Outcomes Measurement Information System) |
| S78 | | HADS term | | HADS (Hospital Anxiety and Depression Scale) |
| S79 | | WHOQOL-BREF term | | WHOQOL-BREF (World Health Organization Quality of Life-BREF) |
| S80 | | DLQI term | | DLQI (Dermatology Life Quality Index) |
| S81 | | AQLQ term | | AQLQ (Asthma Quality of Life Questionnaire) |
| S82 | | PHQ-9 term | | PHQ-9 (Patient Health Questionnaire-9) |
| S83 | | PROMIS Parent Proxy term | | PROMIS Parent Proxy Measures |
| S84 | | HAQ-DI term | | HAQ-DI (Health Assessment Questionnaire Disability Index) |
| S85 | | COPD Assessment Test term | | COPD Assessment Test (CAT) |
| S86 | | Child Health Questionnaire term | | CHQ (Child Health Questionnaire) |
| S87 | | Illness experience term | | PERCIEVED ILLNESS EXPERIENCE |
| S88 | | Health Utility Index term | | Health utility index |
| S89 | | Pediatric quality of life questionnaire term | | Pediatric quality of life questionnaire |
| S90 | | Generic health questionnaire term | | generic health questionnaire |
| S91 | | Africa/country block | | S1 OR S2 OR S3 OR S4 OR S5 OR S6 OR S7 OR S8 OR S9 OR S10 OR S11 OR S12 OR S13 OR S14 OR S15 OR S16 OR S17 OR S18 OR S19 OR S20 OR S21 OR S22 OR S23 OR S24 OR S25 OR S26 OR S27 OR S28 OR S29 OR S30 OR S31 OR S32 OR S33 OR S34 OR S35 OR S36 OR S37 OR S38 OR S39 OR S40 OR S41 OR S42 OR S43 OR S44 OR S45 OR S46 OR S47 OR S48 OR S49 OR S50 OR S51 OR S52 OR S53 OR S54 OR S55 |
| S92 | | HRQoL/PROMs block | | S56 OR S57 OR S58 OR S59 OR S60 OR S61 OR S62 OR S63 OR S64 OR S65 OR S66 OR S67 OR S68 OR S69 OR S70 OR S71 OR S72 OR S73 OR S74 OR S75 OR S76 OR S77 OR S78 OR S79 OR S80 OR S81 OR S82 OR S83 OR S84 OR S85 OR S86 OR S87 OR S88 OR S89 OR S90 |
| Final | | Final combined search | | S91 AND S92 |
| **Cochrane search strategy** | | | | |
| **ID** | **Search** | | | |
| #1 | MeSH descriptor: [Africa] explode all trees | | | |
| #2 | (Africa OR African):ti,ab,kw (Word variations have been searched) | | | |
| #3 | (Algeria):ti,ab,kw (Word variations have been searched) | | | |
| #4 | (Angola):ti,ab,kw (Word variations have been searched) | | | |
| #5 | (Benin):ti,ab,kw (Word variations have been searched) | | | |
| #6 | (Botswana):ti,ab,kw (Word variations have been searched) | | | |
| #7 | ("burkina faso"):ti,ab,kw (Word variations have been searched) | | | |
| #8 | (Burundi):ti,ab,kw (Word variations have been searched) | | | |
| #9 | ("Cabo Verde"):ti,ab,kw (Word variations have been searched) | | | |
| #10 | (Cameroon):ti,ab,kw (Word variations have been searched) | | | |
| #11 | ("Central African Republic"):ti,ab,kw (Word variations have been searched) | | | |
| #12 | (Chad):ti,ab,kw (Word variations have been searched) | | | |
| #13 | (Comoros):ti,ab,kw (Word variations have been searched) | | | |
| #14 | ("Democratic Republic of the Congo" OR DRC):ti,ab,kw (Word variations have been searched) | | | |
| #15 | (Congo):ti,ab,kw (Word variations have been searched) | | | |
| #16 | ("Cote d'Ivoire" OR "Ivory Coast"):ti,ab,kw (Word variations have been searched) | | | |
| #17 | (Djibouti):ti,ab,kw (Word variations have been searched) | | | |
| #18 | (Egypt):ti,ab,kw (Word variations have been searched) | | | |
| #19 | ("Equatorial Guinea"):ti,ab,kw (Word variations have been searched) | | | |
| #20 | (Eritrea):ti,ab,kw (Word variations have been searched) | | | |
| #21 | (Eswatini OR Swaziland):ti,ab,kw (Word variations have been searched) | | | |
| #22 | (Ethiopia):ti,ab,kw (Word variations have been searched) | | | |
| #23 | (Gabon):ti,ab,kw (Word variations have been searched) | | | |
| #24 | (Gambia):ti,ab,kw (Word variations have been searched) | | | |
| #25 | (Ghana):ti,ab,kw (Word variations have been searched) | | | |
| #26 | (Guinea):ti,ab,kw (Word variations have been searched) | | | |
| #27 | ("Guinea-Bissau"):ti,ab,kw (Word variations have been searched) | | | |
| #28 | (Kenya):ti,ab,kw (Word variations have been searched) | | | |
| #29 | (Lesotho):ti,ab,kw (Word variations have been searched) | | | |
| #30 | (Liberia):ti,ab,kw (Word variations have been searched) | | | |
| #31 | (Libya):ti,ab,kw (Word variations have been searched) | | | |
| #32 | (Madagascar):ti,ab,kw (Word variations have been searched) | | | |
| #33 | (Malawi):ti,ab,kw (Word variations have been searched) | | | |
| #34 | (Mali):ti,ab,kw (Word variations have been searched) | | | |
| #35 | (Mauritania):ti,ab,kw (Word variations have been searched) | | | |
| #36 | (Mauritius):ti,ab,kw (Word variations have been searched) | | | |
| #37 | (Morocco):ti,ab,kw (Word variations have been searched) | | | |
| #38 | (Mozambique):ti,ab,kw (Word variations have been searched) | | | |
| #39 | (Namibia):ti,ab,kw (Word variations have been searched) | | | |
| #40 | (Niger):ti,ab,kw (Word variations have been searched) | | | |
| #41 | (Nigeria):ti,ab,kw (Word variations have been searched) | | | |
| #42 | (Rwanda):ti,ab,kw (Word variations have been searched) | | | |
| #43 | ("Sao Tome and Principe"):ti,ab,kw (Word variations have been searched) | | | |
| #44 | (Senegal):ti,ab,kw (Word variations have been searched) | | | |
| #45 | (Seychelles):ti,ab,kw (Word variations have been searched) | | | |
| #46 | ("Sierra Leone"):ti,ab,kw (Word variations have been searched) | | | |
| #47 | (Somalia):ti,ab,kw (Word variations have been searched) | | | |
| #48 | ("South Africa"):ti,ab,kw (Word variations have been searched) | | | |
| #49 | ("South Sudan"):ti,ab,kw (Word variations have been searched) | | | |
| #50 | (Sudan):ti,ab,kw (Word variations have been searched) | | | |
| #51 | (Tanzania):ti,ab,kw (Word variations have been searched) | | | |
| #52 | (Togo):ti,ab,kw (Word variations have been searched) | | | |
| #53 | (Tunisia):ti,ab,kw (Word variations have been searched) | | | |
| #54 | (Uganda):ti,ab,kw (Word variations have been searched) | | | |
| #55 | (Zambia):ti,ab,kw (Word variations have been searched) | | | |
| #56 | (Zimbabwe):ti,ab,kw (Word variations have been searched) | | | |
| #57 | #1 OR #2 OR #3 OR #4 OR #5 OR #6 OR #7 OR #8 OR #9 OR #10 OR #11 OR #12 OR #13 OR #14 OR #15 OR #16 OR #17 OR #18 OR #19 OR #20 OR #21 OR #22 OR #23 OR #24 OR #25 OR #26 OR #27 OR #28 OR #29 OR #30 OR #31 OR #32 OR #33 OR #34 OR #35 OR #36 OR #37 OR #38 OR #39 OR #40 OR #41 OR #42 OR #43 OR #44 OR #45 OR #46 OR #47 OR #48 OR #49 OR #50 OR #51 OR #52 OR #53 OR #54 OR #55 OR #56 | | | |
| #58 | MeSH descriptor: [Quality of Life] explode all trees | | | |
| #59 | MeSH descriptor: [Health Status] explode all trees | | | |
| #60 | MeSH descriptor: [Pain Measurement] explode all trees | | | |
| #61 | MeSH descriptor: [Outcome Assessment, Health Care] explode all trees | | | |
| #62 | MeSH descriptor: [Patient Reported Outcome Measures] explode all trees | | | |
| #63 | MeSH descriptor: [Psychometrics] explode all trees | | | |
| #64 | ("health related quality of life" OR HRQoL OR "quality of life" OR QoL):ti,ab,kw (Word variations have been searched) | | | |
| #65 | ("pain measurement" OR "pain assessment" OR "pain scale"):ti,ab,kw (Word variations have been searched) | | | |
| #66 | ("patient reported outcome measure" OR "patient reported outcome measures" OR PROM OR PROMs OR "patient outcome assessment" OR "preference based measure" OR "preference based assessment" OR "generic health profile measure" OR "disease health status measure" OR "psychometric measure" OR "psychometric measures"):ti,ab,kw (Word variations have been searched) | | | |
| #67 | (EuroQoL OR "European quality of life questionnaire" OR EQ-5D OR EQ-5D-5L OR EQ-5D-Y OR EQ-5D-Y-5L OR EQ-5D-Y-3L OR "EuroQol 5-Dimension"):ti,ab,kw (Word variations have been searched) | | | |
| #68 | ("Short Form-36" OR "short form 36" OR SF-36):ti,ab,kw (Word variations have been searched) | | | |
| #69 | ("functional assessment" OR "functional assessment of cancer therapy" OR FACT):ti,ab,kw (Word variations have been searched) | | | |
| #70 | ("QLQ-C30" OR "Quality of Life Questionnaire-Core 30" OR PROMIS OR "Patient-Reported Outcomes Measurement Information System" OR HADS OR "Hospital Anxiety and Depression Scale" OR WHOQOL-BREF OR "World Health Organization Quality of Life-BREF" OR DLQI OR "Dermatology Life Quality Index" OR AQLQ OR "Asthma Quality of Life Questionnaire" OR PHQ-9 OR "Patient Health Questionnaire-9"):ti,ab,kw (Word variations have been searched) | | | |
| #71 | ("PROMIS Parent Proxy Measures" OR HAQ-DI OR "Health Assessment Questionnaire Disability Index" OR "COPD Assessment Test" OR CAT OR CHQ OR "Child Health Questionnaire" OR "perceived illness experience" OR "Health Utility Index" OR "Pediatric Quality of Life Questionnaire" OR "generic health questionnaire"):ti,ab,kw (Word variations have been searched) | | | |
| #72 | #58 OR #59 OR #60 OR #61 OR #62 OR #63 OR #64 OR #65 OR #66 OR #67 OR #68 OR #69 OR #70 OR #71 | | | |
| #73 | #57 AND #72 | | | |

|  |  |
| --- | --- |
| 1996 | 1 |
| 1997 | 1 |
| 1998 | 1 |
| 2001 | 2 |
| 2002 | 1 |
| 2003 | 2 |
| 2004 | 8 |
| 2005 | 5 |
| 2006 | 12 |
| 2007 | 12 |
| 2008 | 13 |
| 2009 | 23 |
| 2010 | 26 |
| 2011 | 27 |
| 2012 | 26 |
| 2013 | 50 |
| 2014 | 49 |
| 2015 | 35 |
| 2016 | 54 |
| 2017 | 48 |
| 2018 | 52 |
| 2019 | 45 |
| 2020 | 59 |
| 2021 | 64 |
| 2022 | 74 |
| 2023/24 | 41 |

**Figure S1:** Illustrates the annual publication trends of HRQoL studies in Africa.

**Table S2:** The geographic distribution of HRQoL study coverage across African countries

| **Country** | **Number of studies** | **Percentage (%)** |
| --- | --- | --- |
| Nigeria | 159 | 21.8 |
| Ethiopia | 108 | 14.8 |
| South Africa | 106 | 14.5 |
| Egypt | 54 | 7.4 |
| Uganda | 47 | 6.4 |
| Kenya | 34 | 4.7 |
| Ghana | 31 | 4.2 |
| Morocco | 30 | 4.1 |
| Tunisia | 27 | 3.7 |
| Tanzania | 21 | 2.9 |
| Cameroon | 13 | 1.8 |
| Malawi | 12 | 1.6 |
| Zimbabwe | 10 | 1.4 |
| Sudan | 9 | 1.2 |
| Rwanda | 7 | 1.0 |
| Botswana | 4 | 0.6 |
| Congo | 4 | 0.6 |
| Burkina Faso | 3 | 0.4 |
| Zambia | 3 | 0.4 |
| Guinea | 2 | 0.3 |
| Madagascar | 2 | 0.3 |
| Senegal | 2 | 0.3 |
| Togo | 2 | 0.3 |
| Algeria | 1 | 0.1 |
| Benin | 1 | 0.1 |
| Cote D'ivoire | 1 | 0.1 |
| Lesotho | 1 | 0.1 |
| Mauritius | 1 | 0.1 |
| Namibia | 1 | 0.1 |
| Sierra Leone | 1 | 0.1 |
| Swaziland | 1 | 0.1 |
| Multiple countries (at least two or more from the following list): Algeria, Benin, Botswana, Ghana, Kenya, Lesotho, Malawi, Morocco, Mozambique, Namibia, Nigeria, South Africa, Swaziland, Tanzania, Togo, Tunisia, Uganda, Zambia, Zimbabwe | 33 | 4.5 |

**Table S3:** Distribution of reported information across EQ-5D versions in studies utilizing the EQ-5D instruments (n=96).

| **Type of information reported** | **EQ-5D-3L** | **EQ-5D-5L** | **EQ-VAS only** | **EQ-6D** | **Not reported** |
| --- | --- | --- | --- | --- | --- |
| Descriptive profile | 8 | 1 | 0 | 1 | 0 |
| Descriptive profile and EQ-VAS | 13 | 3 | 0 | 0 | 0 |
| Descriptive profile and index | 6 | 2 | 0 | 0 | 0 |
| Descriptive profile, index and EQ-VAS | 12 | 17 | 0 | 0 | 2 |
| EQ-VAS | 6 | 0 | 4 | 0 | 0 |
| Index | 8 | 0 | 0 | 0 | 2 |
| Index and EQ-VAS | 6 | 3 | 0 | 0 | 1 |
| Not reported | 0 | 0 | 0 | 0 | 1 |
